# Expression and purification of soluble recombinant β-lactamases using *Escherichia coli* as expression host and pET-28a as cloning vector

**DOI:** 10.1186/s12934-022-01972-5

**Published:** 2022-11-23

**Authors:** Lele Li, Hui Li, Qingwu Tian, Baosheng Ge, Xiaotong Xu, Yuanyuan Chi, Huaizhi Zhao, Yanfei Liu, Nan Jia, Tingting Zhou, Yuanqi Zhu, Yusun Zhou

**Affiliations:** 1 Department of Clinical Laboratory, The Affiliated Hospital of Qingdao University, Qingdao, 266000 Shandong China; 2 State Key Laboratory of Heavy Oil Processing and Center for Bioengineering and Biotechnology, China University of Petroleum (East China), Qingdao, 266580 Shandong China; 3 Department of Laboratory Medicine, The Fourth Affiliated Hospital Zhejiang University School of Medicine, Yiwu, 322000 Zhejiang China; 4 Department of Pediatric Emergency, The Affiliated Hospital of Qingdao University, Qingdao, 266000 Shandong China; 5 Qingdao Women and Children’s Hospital, Qingdao, 266034 Shandong China

**Keywords:** Recombinant β-lactamases, Soluble protein expression, Protein affinity purification, Recombinant pET-28a plasmids, *Escherichia coli* BL21 (DE3)

## Abstract

**Background:**

Due to its high expression capability, recombination of *Escherichia coli* and pET vector has become the bioengineering preferred expression system. Because β-lactamases mediate bacterial antimicrobial resistance, these enzymes have a substantial clinical impact. Using the *E. coli* expression system, several kinds of β-lactamases have been produced. However, previous studies have been focused on characterizing target β-lactamases, and the effects of cultivation and induction conditions on the expression efficiency of target enzymes were not addressed.

**Results:**

Using pET-28a as the cloning vector and *E. coli* BL21(DE3) as the expression host, this study originally elucidated the effects of IPTG concentration, culture temperature, induction time, and restriction sites on recombinant β-lactamase expression. Moreover, the effects of the target protein length and the 6 × His-tag fusion position on enzyme purification were also explored, and consequently, this study yielded several important findings. (i) Only the signal peptide–detached recombinant β-lactamase could exist in a soluble form. (ii) Low-temperature induction was beneficial for soluble β-lactamase expression. (iii) The closer to the *rbs* the selected restriction site was, the more difficult it was to express soluble β-lactamase. (iv) The short-chain recombinant protein and the protein with His-tag fused at its C-terminus showed high affinity to the Ni^2+^ column.

**Conclusions:**

Based on our findings, researchers can easily design an effective program for the high production of soluble recombinant β-lactamases to facilitate other related studies.

## Background

Recombinant DNA technology of *Escherichia coli* offers several advantages for high-level expression and scalable production of proteins of interest by relatively inexpensive procedure [1]. Such process of recombinant protein production requires: (i) selection of an appropriate cloning vector, the target gene and a competent host for expression generation; (ii) generation of a stable and high-yielding recombinant clone; (iii) optimization of a consistent and scalable fermentation process for protein expression; and (iv) purification of the fusion protein using a suitable and convenient affinity purification system [2].

IPTG (isopropyl-*β*-*D*-thio-galactopyranoside)-inducible pET plasmids (with the *T7* as promoter, recognized by T7 RNA polymerase, and the *lac* as operator) and operon-reconstructed derivatives, such as cumate (*p*-isopropylbenzoate)-inducible pNEW plasmids (with the *T5* as promoter, recognized by *E. coli* RNA polymerase, and the *cmt* as operator), are two vector categories for target gene cloning [3]. The target gene–inserted vector is transformed into a competent *E. coli* strain (with the B-series strains as the extensively used hosts [4]) for high-level expression of recombinant proteins. Conducting the fermentation process in an intelligent, large-scale, and controllable fermenter facilitates simple scale-up and cost-effective production of target proteins [5]. Fusing the recombinant protein with functional tags (such as His, SUMO, and Fh8) at its N- or C-terminus is convenient for efficient affinity purification and enhances the soluble expression of the protein [6].

Many therapeutic proteins and industrial enzymes have been produced for clinical and academic applications using *E. coli* expression systems, accounting for approximately 30% of the currently approved recombinant proteins [7–9]. It is critical to select a suitable strain for heterologous gene expression based on the characteristics of the available strains and target proteins. For example, *E. coli* K-12 strains and their derivatives produce high levels of acetate, which is detrimental to cell growth and protein expression; thus, these strains are more suitable for propagating recombinant DNA library clones [7]. *E. coli* B strains, however, exhibit low acetate accumulation and are commonly used for target protein expression [4]. The *E. coli* BL21 (DE3) strain has many preferred characteristics that support heterologous gene expression, such as defects in Lon protease (degrading foreign proteins in the cytoplasm) and OmpT protease (degrading extracellular proteins) genes and a *T7* RNA polymerase gene under the control of the *lacUV5* promoter (recognized by *E. coli* RNA polymerase); while the leaky expression arisen from T7 RNA polymerase is readily mitigated by the addition of 1% (w/v) glucose to the cultivation medium [10]. To date, various derivatives of BL21 (DE3), such as BL21 (DE3)pLysS, Lemo21(DE3), Origami, and SHuffle, have been designed for preventing leaky expression of T7 RNA polymerase, and increasing soluble protein expression, enhancing disulfide bond formation, and promoting the correct folding of target proteins [11].

The pET vectors possess the potent *T7* promoter and are considered the first choice for cloning and protein expression. Due to high expression capability, combination of pET vectors harbored by *E. coli* BL21 (DE3) has become the biopharmaceutical and industrial preferred expression system [11]. Heterologous gene expression can be simply regulated from low to high levels in the aforementioned system by adjusting the IPTG concentration in the cultivation medium, which, to a certain extent, could prevent negative effects on host cells caused by heterologous protein overexpression in the case of toxicity or insolubility issues [12]. The N-terminus of most pET vectors is the preferable location for including additional tags, such as the 6 × His affinity tag and the NusA hydrotropy tag [7]. Upon transcription, these tags will be incorporated into the target proteins, allowing their easy recovery and purification. In addition, prolonging induction at a low temperature and lowering the inducer concentration can increase the solubility of recombinant proteins, reduce the formation of inclusion bodies, and maintain the structure and stability of target proteins [13].

β-Lactamases are bacterial enzymes with a great clinical impact as they mediate β-lactam antibiotic resistance in many Gram-negative bacteria. Developing new strategies for producing recombinant β-lactamases facilitates structure-function relationship studies. In the last decade, using pET plasmids as cloning vectors and *E. coli* BL21 (DE3) as the expression host, several kinds of β-lactamases have been produced, including IMP-13 [14], OXA-17 [15], and OXA-205 [16]. These studies were focused on acquiring and characterizing the target β-lactamases, whereas the effects of the pET vector restriction sites selected and the cultivation and induction conditions used on the expression efficiency of target enzymes (especially their soluble fractions) have not been addressed. Herein, in this study, the most prevalent SHV- and TEM-type β-lactamases were evaluated using pET-28a as the cloning vector and *E. coli* BL21 (DE3) as the expression host to systematically investigate the effects of several important parameters on enzyme production. Accordingly, the homogeneity of the purified enzymes was confirmed by SDS-PAGE and MALDI-TOF MS analysis.

This study aimed to help researchers design an appropriate approach for the high production of soluble recombinant β-lactamases to facilitate other related studies. These studies involve a progressive design, allowing the expression and characterization of soluble new-found or sequence-altered β-lactamases, rapid exploration of the enzymes' hydrolyzing profiles on β-lactam antibiotics, further optimization of extraction conditions for selectively separating the target enzymes from bacteria, and finally a novel testing technique for quickly guiding clinical antimicrobial medication will be established.

## Materials and methods

### Genetic manipulations

Two broad-spectrum β-lactamases, SHV-1 and TEM-1, encoded by the *bla*_SHV-1_ and *bla*_TEM-1_ genes, respectively, were chosen as expression targets in this study. Nucleotide sequences of the two genes and their coded amino acid sequences were retrieved from the National Center for Biotechnology Information (NCBI) Nucleotide dataset (https://ncbi.nlm.nih.gov/nuccore/; GenBank accession nos. GQ407127 and EF035581) and UniProtKB (https://www.expasy.org/resources/uniprotkb; accession nos. P0AD64 and P62593), respectively. Both genes are 861 bp in length, each encoding a 286-amino acid protein. The molecular weight (M_w_) of SHV-1 is 31,224 Da, and it has a signal peptide comprising 21 amino acid residues at its N-terminus. The M_w_ of TEM-1 is 31,515 Da, and its signal peptide is an N-terminal sequence comprising 23 amino acids.

**Fig. 1:**
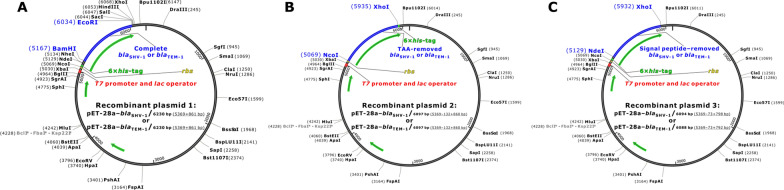
Three kinds of recombinant pET-28a plasmids for each expression target (SHV-1 and TEM-1) (**A**, **B**, and **C**: recombinant plasmid 1, 2, and 3, respectively)

Gene recombinations were submitted to Sangon Biotech Co., Ltd. (Shanghai, China) for completion. The three recombinant pET-28a plasmids generated per expression target are presented in Table 1 and Fig. 1. (i) Recombinant plasmid 1: The complete *bla*_SHV-1_ or *bla*_TEM-1_ was synthesized and inserted between the *Bam*HI and *Eco*RI restriction sites, producing the recombinant plasmids pET-28a–*bla*_SHV-1_/6230 bp and pET-28a–*bla*_TEM-1_/6230 bp (Fig. 1a). (ii) Recombinant plasmid 2: The TAA-removed *bla*_SHV-1_ or *bla*_TEM-1_ was inserted between the *Nco*I and *Xho*I restriction sites, producing the pET-28a–*bla*_SHV-1_/6097 bp and pET-28a–*bla*_TEM-1_/6097 bp plasmids (Fig. 1b). (iii) Recombinant plasmid 3: The signal peptide coding region–removed *bla*_SHV-1_ or *bla*_TEM-1_ was inserted between the *Nde*I and *Xho*I sites, producing the pET-28a–*bla*_SHV-1_/6094 bp and pET-28a–*bla*_TEM-1_/6088 bp plasmids (Fig. 1c).

**Table 1:** Three kinds of recombinant pET-28a plasmids and the corresponding recombinant proteins for each expression target

Expression targets	Recombinant plasmids	Recombinant proteins	M_w_ (Da) of the expressed proteins
SHV-1	1. pET-28a–*bla*_SHV-1_/6230 bp	1a. (MGSSHHHHHH…RGS)^a^(SHV-1)	34,768
		1b. Signal peptide–detached SHV-1	28,874
	2. pET-28a–*bla*_SHV-1_/6097 bp	2a. (MA)^a^(SHV-1)(LEHHHHHH)^a^	32,491
		2b. (Signal peptide–detached SHV-1)(LEHHHHHH)^a^	29,939
	3. pET-28a–*bla*_SHV-1_/6094 bp	3. (MGSSHHHHHH…SHM)^a^( Signal peptide–removed SHV-1)	31,169
TEM-1	1. pET-28a–*bla*_TEM-1_/6230 bp	1a. (MGSSHHHHHH…RGS)^a^(TEM-1)	35,059
		1b. Signal peptide–detached TEM-1	28,907
	2. pET-28a–*bla*_TEM-1_/6097 bp	2a. (MA)^a^(TEM-1)(LEHHHHHH)^a^	32,783
		2b. (Signal peptide–detached TEM-1)(LEHHHHHH)^a^	29,972
	3. pET-28a–*bla*_TEM-1_/6088 bp	3. (MGSSHHHHHH…SHM)^a^( Signal peptide–removed TEM-1)	31,202

^a^: fusion peptide; HHHHHH: 6 × His-tag

The DNA sequences of the recombinant plasmids were confirmed (Fig. 1) and transformed into *E. coli* BL21 (DE3), and the transformants were selected on LB agar plates containing 60 μg/mL of kanamycin. The cultivated monoclonal transformants were further verified by sequencing and stored in 50% (v/v) glycerine for the subsequent expression of recombinant SHV-1 and TEM-1.

### Optimization of expression conditions

Induced expression of recombinant β-lactamases in 50 mL or 3 L of LB medium containing 60 μg/mL of kanamycin was conducted in conical flasks and a fermenter, respectively. The former volume was used for optimizing the expression conditions, and the latter was used for expressing recombinant enzymes under the established optimal conditions. First, we explored the effects of IPTG concentration, culture temperature, and induction time on enzyme expression using recombinant plasmid 1–transformed *E. coli* BL21 (DE3) as the expression host (host 1).

After activation and amplification, 500 μL of host 1 cells was added to each of four conical flasks containing 50 mL of LB medium and cultivated in an incubator at 37 °C under continuous circular oscillation (180 rpm). A different concentration of IPTG (0, 0.25, 0.5, and 1 mM) was added to each tube after 2 h of incubation. IPTG was added in mid-log phase of cell growth, with an optical density (OD_600_) of approximately 0.83. Five induction time nodes (2, 4, 8, 16, and 32 h) were chosen, and 1 mL of each cell suspension was collected at each time node for protein extraction (both soluble and insoluble fractions) and SDS-PAGE analysis.

**Fig. 2:**
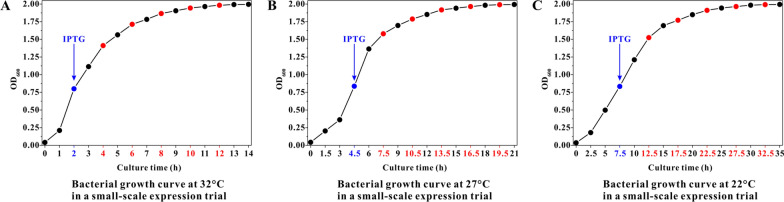
Growth curves of host 1 cells separately cultivated at 32 (**A**), 27 (**B**), and 22 °C (**C**) in small-scale expression trials (blue-marked: IPTG added to the culture systems; red-marked: induction time nodes)

Another three culture systems were separately cultivated at 32, 27, and 22 °C, in which 0.5 mM IPTG was added in mid-log phase of cell growth (OD_600_ ≈ 0.83). Three batches of induction time nodes were set for the three culture systems, including 2, 4, 6, 8, and 10 h for the cultured at 32 °C, 3, 6, 9, 12, and 15 h for the cultured at 27 °C, and 5, 10, 15, 20, and 25 h for the cultured at 22 °C (Fig. 2). At the last induction time node (10, 15, and 25 h) for each culture temperature, the values of OD_600_ reached 1.99 and remained constant despite the culture time going. Cell suspensions collected at the final time points were processed by BCA (bicinchoninic acid) method to obtain soluble and insoluble protein concentrations.

In the next step, under the optimal culture temperature (22 °C) and induction conditions (0.5 mM IPTG induction for 25 h), using recombinant plasmid 2 and 3–transformed *E. coli* BL21 (DE3) as the expression hosts (host 2 and 3), the effects of restriction sites on enzyme expression were investigated by SDS-PAGE analysis and BCA protein quantification.

### Expression and purification of recombinant β-lactamases

#### Expression

Induced expression of recombinant protein 3 was conducted in Minifors 2 bench-top fermenter (INFORS HT, Switzerland) controlled by EVE^®^, a bioprocess platform software. First, 1.5 mL of activated host 3 cells was added to 150 mL of LB medium containing 60 μg/mL of kanamycin, cultivated overnight at 37 °C, and injected into the fermenter containing 3 L of sterile LB medium supplemented with 60 μg/mL of kanamycin. Host 3 cells were then cultured at 37 °C for 1 h; then, the temperature was reduced to 22 °C in 0.5 h and kept steady for another 0.5 h until the OD_600_ reached 0.83. Subsequently, 0.5 mM IPTG was injected into the culture system to induce protein expression for 25 h, during which 300 mL of 10 × LB medium containing 10% (w/v) glucose instead of 10% (w/v) NaCl was pumped into the fermenter at a rate of 8%·3.5 mL/min after 1.5 h of induction. Over the entire incubation process, a pH of 7 and a dissolved oxygen content of 40% were maintained by the automatic adjustment of acid-base pumping, airflow velocity, and motor stirring speed.

#### Adsorption

After induction, host 3 cells were harvested by centrifugation at 7500 rpm for 10 min at 4 °C, washed twice with PBS (pH 8.2), and resuspended homogeneously in 160 mL of PBS. Lysozyme (0.1 g) and 1 mM PMSF (phenylmethylsulphonyl fluoride) were added for cell breakage under high pressure. Cell breakage was conducted at 4 °C for 4–5 times, the collected lysis solution was centrifuged at 10,000 rpm for 30 min at 4 °C, and the clarified supernatant was obtained. The protein supernatant was filtered through 0.45 μm hydrophilic membranes and loaded at 2 mL/min onto two tandem HisTrap HP columns (each prepacked with 5 mL of high-performance Ni^2+^ sepharose medium) (Cytiva, USA) previously equilibrated with 5 column volumes of buffer A (0.4 M NaCl dissolved in PBS, pH 8.2). The flow-through fraction was collected for protein purification validation.

#### Purification

The two Ni^2+^ columns capturing soluble protein 3 were separately connected to an ÄKTA pure system (Cytiva, USA) controlled by UNICORN™ software for enzyme purification. The mobile phase comprised two parts of buffer A and B (0.5 M imidazole dissolved in buffer A, pH 8.2) with a flow rate of 2 mL/min, and the monitoring wavelength was set at 280 nm. First, the Ni^2+^ column was washed with 5 column volumes of buffer A to elute unadsorbed proteins until the signal response was balanced with the instrument baseline. Then, the proportion of buffer B in the mobile phase was increased to 2% and 6% to elute non-target proteins. Elution solutions were collected when the protein signal could be detected. The proportion of buffer B was further increased to 50%, and the purified protein 3 was eluted and collected.

#### Desalination

Finally, the purified protein 3 saline solution was desalted using a HiTrap Desalting column (prepacked with 5 mL of superfine sephadex G-25 material) (Cytiva, USA). The column was connected to the ÄKTA system and equilibrated with 5 column volumes of mobile phase (deionized water). For desalting, the flow rate was maintained at 2 mL/min, and the monitoring wavelength was set at 280 nm. During desalting, the instrument pump was paused, and 1 mL of protein 3 saline solution was injected into the column. Then, the ÄKTA instrument was restarted and the desalted protein 3 aqueous solution was collected. After eluting salt from the column, the desalting procedure above was repeated until protein desalination was completed.

### SDS-PAGE analysis and protein measurement

The proteins extracted in the optimization process and the soluble proteins obtained from the purification process were analyzed by SDS-PAGE. Gel preparation and electrophoresis were conducted as described by Solarbio Life Sciences Co., Ltd. (Beijing, China). Before electrophoresis, 80 μL of protein solution or suspension and 20 μL of 5 × loading buffer (containing SDS and DTT (dithiothreitol)) were mixed by vortexing and heated to 100 °C in a metal bath (ThermoCell Dry Bath HB-100, Bioer Technology Co., Ltd., Hangzhou, China) for protein denaturation. After 10 min of denaturation, the mixture was centrifuged at 13,000 rpm for 5 min and a clarified supernatant was obtained, in which soluble or insoluble proteins were present in a dissolved denatured form. Then, 10 μL of supernatant and 5 μL of marker (11–180 kDa) were loaded for protein electrophoresis at 80 V for 1 h and 120 V for a subsequent 1 h. A Solarbio SDS-PAGE Gel Kit was used, comprising a stacking and separating gel containing 5% and 10% (w/v) acrylamide, respectively. After electrophoresis, the separating gel was taken out and stained for 1 h, and the distinct protein bands were analyzed.

Protein concentrations were determined by BCA method using BSA (bovine serum albumin) as a standard with an assay kit from Sangon Biotech. The calibration curve was plotted with a series of BSA concentrations as the X-axis and the reaction product absorbance at 562 nm as the Y-axis, which had a good linearity in the range of 0–500 μg/mL with R^2^ > 0.99. The PBS-diluted soluble protein solution and insoluble protein suspension were both submitted to BCA method for protein concentration determination.

## Results and discussion

### IPTG concentration

**Fig. 3:**
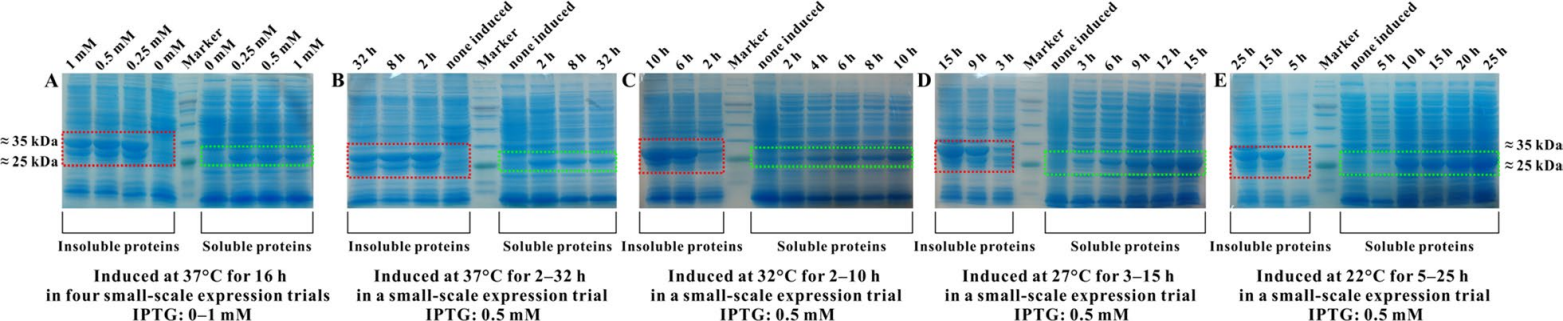
Effects of IPTG concentration (**A**), culture temperature (**B**–**E**), and induction time (**C**–**E**) on recombinant SHV-1 (protein 1) expression (The same results were also obtained in the TEM-1 heterologous expression)

Regulation of inducer concentration is critical for recombinant protein expression [17, 18]. High concentration of inducer can have toxic effects on host cells, thus preventing protein expression. Low inducer concentration can lead to insufficient induction, thus lowering protein expression efficiency. In the literature, IPTG concentrations between 0 and 1 mM appear safe for *E. coli* growth [7, 11]. Thus, in this study, using recombinant SHV-1 (protein 1) as expression target, four concentrations of 0, 0.25, 0.5, and 1 mM IPTG were evaluated for expression induction. As seen in Fig. 3a, when IPTG was absent, no recombinant protein was produced, and there were no significant differences in protein expression induction among the three IPTG concentrations. Thus, a concentration of 0.5 mM IPTG was used for the following experimentations. In addition, two fractions (protein 1a and 1b) of recombinant SHV-1 were obtained, as shown in Fig. 3a. The complete-sequence protein 1a was only present in the form of insoluble inclusion bodies, whereas the signal peptide–detached protein 1b appeared in soluble and insoluble forms. The same results were also obtained in the TEM-1 heterologous expression. Thus, it was concluded that only the recombinant protein with the signal peptide deleted could fold correctly and form a soluble three-dimensional structure.

### Culture temperature and induction time

As seen in Fig. 3b, prolonging the induction time did not increase recombinant protein expression when host cells were cultivated at 37 °C. This finding was mainly attributed to by-product acetate accumulation in the culture medium due to fast cell metabolism at higher temperature, which caused a low protein expression level [1, 13]. Fortunately, acetate accumulation could be diminished by reducing the metabolic rate of host cells at lower temperature. As shown in Fig. 3c–e, recombinant protein expression increased as the culture temperature was decreased, and a higher concentration of soluble target proteins was produced as induction time increased. In addition, cell harvesting of 1 mL of culture suspension was conducted prior to the stationary phase of cell growth (i.e., at the last induction time node of each culture temperature) for protein extraction and concentration determination. As shown in Table 2, under low-temperature cultivation conditions, more soluble and fewer insoluble proteins were obtained as the culture temperature dropped. These results are consistent with previous studies in which low-temperature induction was revealed to enhance correct protein folding, increase protein solubility and stability, and prevent inclusion body formation [19, 20]. The aforementioned findings were also observed with the TEM-1 heterologous expression.

**Table 2:** Concentrations of proteins (recombinant SHV-1 (protein 1)) extracted from 1 mL of cell suspensions collected at the last induction time nodes in three parallel small-scale expression trials

Culture temperatures	Induction time nodes	Soluble proteins (μg/mL)	Insoluble proteins (μg/mL)
32 °C	10 h	3958	6200
27 °C	15 h	4316	4453
22 °C	25 h	8532	3582

### Restriction sites

**Fig. 4:**
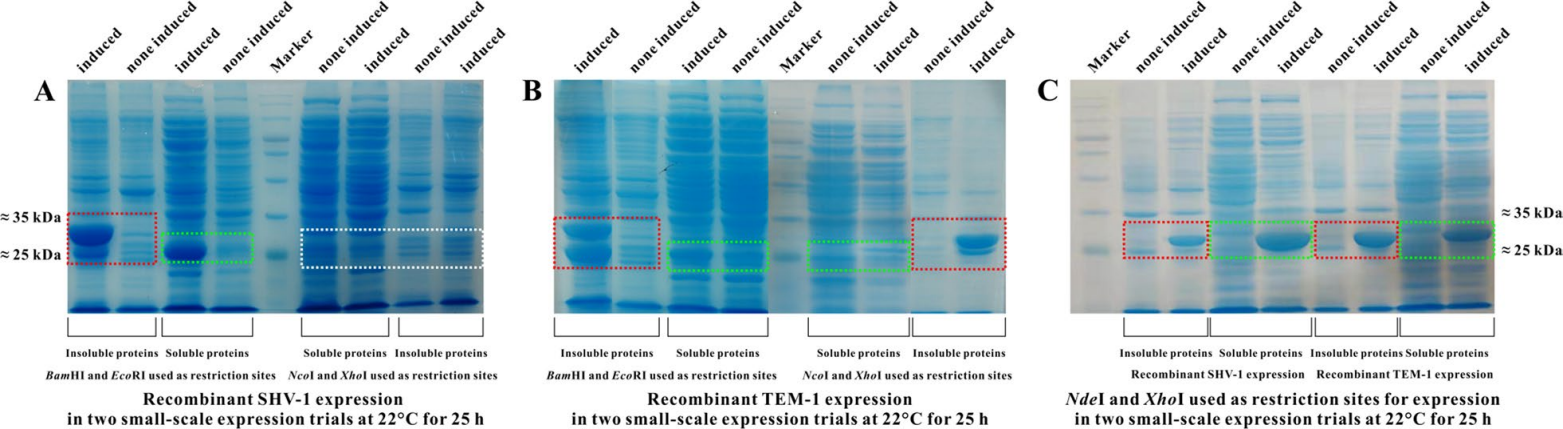
Effects of restriction sites on recombinant β-lactamase expression (**A**: recombinant SHV-1 (protein 1 and 2) expression using *Bam*HI/*Eco*RI and *Nco*I/*Xho*I as restriction sites, respectively; **B**: recombinant TEM-1 (protein 1 and 2) expression using *Bam*HI/*Eco*RI and *Nco*I/*Xho*I as restriction sites, respectively; and **C**: the protein 3 (recombinant SHV-1 and TEM-1) expression using *Nde*I/*Xho*I as restriction sites)

The selection of restriction sites for inserting target genes is another important factor affecting recombinant protein expression. As shown in Fig. 4a, under the optimal expression conditions, no recombinant SHV-1 (protein 2) was expressed when the *Nco*I and *Xho*I restriction sites were used. This effect can be attributed to two potential reasons, one of which is that the formation of a secondary structure at the translation initiation site of *bla*_SHV-1_ that coupled with the *rbs* target region on the ribosome prevented translation proceeding [11]. The second potential reason is the translation repression due to an electrostatic interaction between the positively charged nascent peptide of the fusion protein and the negatively charged ribosomal exit tunnel [21]. Although recombinant TEM-1 (protein 2) expression proceeded as expected, the soluble protein 2b production was greatly diminished vs. that of protein 1b (Fig. 4b). Therefore, we hypothesized that the closer to the *rbs* on pET vector the selected restriction site was, the more difficult it would be for the soluble recombinant β-lactamase to be expressed. The protein 3 expression results supported this hypothesis; when the *Nde*I and *Xho*I restriction sites were used, more target proteins were expressed than observed with protein 2 (Fig. 4c).

### His-tag

**Fig. 5:**
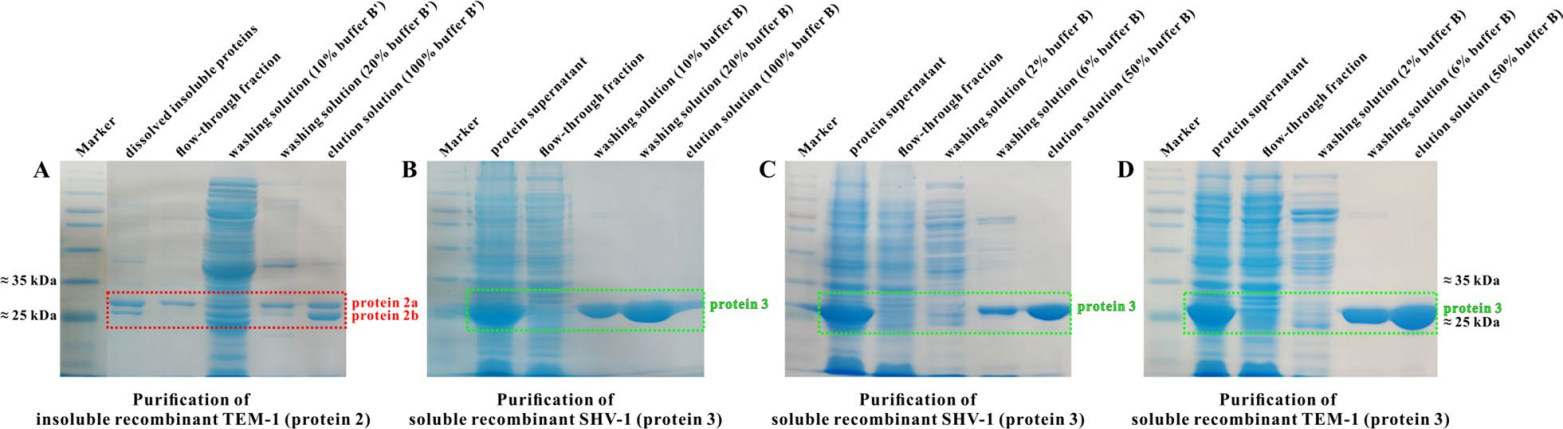
Effects of target protein length (**A**: purification of insoluble recombinant TEM-1 (protein 2) using buffer A' and B' (both containing 4 M urea) as the mobile phase) and 6 × His-tag fusion position (**B**–**D**: purification of protein 3 (soluble recombinant SHV-1 and TEM-1) using buffer A and B as the mobile phase) on the purification efficiency of recombinant β-lactamases

The 6 × *his*-tag on pET-28a vector can simplify protein purification using the Ni^2+^ affinity column by adding 6 × His-tag to recombinant proteins after transcription. Due to its small size, the 6 × His-tag can easily be internalized into the molecular structure, reducing the solubility of target proteins and their affinity to the Ni^2+^ column [11, 22]. Thus, only fusion proteins with His-tag exposed on the outside (i.e., added to the N- or C-terminus of target proteins) can be recovered and purified. In this study, to minimize the impact of His-tag on protein solubility, all recombinant proteins were designed with the 6 × His-tag only fused at their one terminus. The protein purification results presented in Fig. 5 indicate that the length of the target proteins and the fusion position of the His-tag influenced protein affinity to the Ni^2+^ column.

#### Protein length

His-tag chelation of Ni^2+^ can proceed even in the presence of high concentrations of chaotropic agents, such as 8 M urea, which is usually used to denature and dissolve insoluble proteins [23, 24]. Therefore, in this study, the inclusion bodies of recombinant TEM-1 (protein 2) dissolved in buffer A'' (containing 8 M urea) were purified using buffer A' and B' (both containing 4 M urea) as the mobile phase. As seen in Fig. 5a, in dissolved inclusion bodies, the mass of signal peptide–detached protein 2b was less than that of complete-sequence protein 2a, whereas after purification, the mass of protein 2b was higher than that of protein 2a, indicating that the short-chain protein 2b exhibited a stronger affinity to the Ni^2+^ column than the long-chain protein 2a, making more protein 2a eluted in the washing step.

#### Fusion position

The purification procedure for insoluble recombinant TEM-1 (protein 2) was also applied to soluble protein 3. The purification results are shown in Fig. 5b and recombinant SHV-1 is presented as an example because the same results were also obtained with TEM-1. As seen in it, most of protein 3 was eluted when 10–20% buffer B was used as a washing solution, indicating that the recombinant proteins with His-tag fused at their N-terminus showed a much lower affinity to the Ni^2+^ column than proteins with His-tag fused at the C-terminus. Thus, to enhance the purification efficiency of soluble protein 3, the proportion of buffer B in washing solution was relegated to 2–6% for non-target protein elution, and then increased to 50% for purified protein 3 elution and collection (Fig. 5c and d).

### MS confirmation

**Fig. 6:**
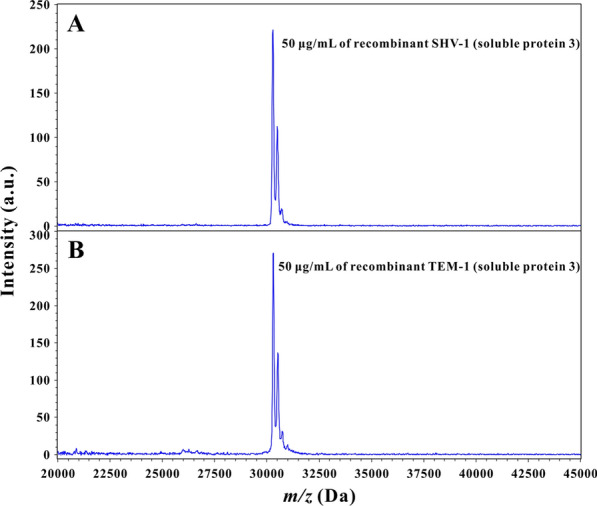
MS confirmation of the purified recombinant β-lactamases (soluble protein 3) (**A**: soluble recombinant SHV-1; and **B**: soluble recombinant TEM-1)

After purification and desalination, 15–20 mL of purified protein 3 aqueous solution was obtained with a concentration of 2500–5000 μg/mL of recombinant β-lactamase. In addition to verification by SDS-PAGE analysis, the purified protein 3 was confirmed by MALDI-TOF MS. For MS measurement, 2 μL of protein 3 aqueous solution (50 μg/mL) was applied onto a single spot on a 96-spot polished steel MALDI target plate. After air drying, the sample was overlaid with 2 μL of matrix solution (a saturated solution of sinapinic acid in 50% acetonitrile and 0.1% trifluoroacetic acid [25]). The matrix/sample spot was allowed to crystallize at room temperature and submitted to a MALDI-TOF mass spectrometer (Bruker Daltonics GmbH) for spectrum acquisition under a positive linear ion mode within *m/z* range from 20,000 to 45,000 Da. As shown in Fig. 6, clearly visible mass peaks of recombinant SHV-1 (a) and TEM-1 (b) and a smooth baseline were present in the spectra with no appearances of other proteins. Thus, the MS measurement results confirmed the homogeneity of the purified recombinant β-lactamases.

## Conclusions

In this study, using SHV-1 and TEM-1 as expression targets, pET-28a as the cloning vector, and *E. coli* BL21(DE3) as the expression host, we systematically investigated the effects of IPTG concentration, culture temperature, induction time, and restriction sites on recombinant β-lactamase expression. Moreover, the effects of the target protein length and the 6 × His-tag fusion position on enzyme purification were also explored, and so several important findings were uncovered. (i) Only the signal peptide–detached recombinant β-lactamase could fold correctly and take on a soluble three-dimensional structure. (ii) Low-temperature induction was beneficial for soluble recombinant β-lactamase expression, and more soluble enzymes were produced as the induction time increased. (iii) The closer to the *rbs* on pET vector the selected restriction site was, the more difficult it was to obtain soluble recombinant β-lactamase expression. (iv) The short-chain recombinant protein and the protein with His-tag fused at its C-terminus showed a much stronger affinity to the Ni^2+^ column than the long-chain protein and the protein fused with His-tag at the N-terminus. These findings allow researchers to design an effective program for the high production of soluble recombinant β-lactamases, upon which a series of functional materials can be prepared using the target enzymes as templates, allowing simultaneous selective separation of multiple β-lactamases with typical epidemiological significance from bacteria, creating quick guidance for clinical antimicrobial medication according to their hydrolyzing profiles of the detected β-lactamases.

## Data Availability

All data generated or analyzed during this study are included in this published article.
